# OX40L induces helper T cell differentiation during cell immunity of asthma through PI3K/AKT and P38 MAPK signaling pathway

**DOI:** 10.1186/s12967-018-1436-4

**Published:** 2018-03-20

**Authors:** Li Huang, Meijuan Wang, Yongdong Yan, Wenjing Gu, Xinxing Zhang, Jiahong Tan, Huiming Sun, Wei Ji, Zhengrong Chen

**Affiliations:** Department of Pediatric Pulmonology, Children’s Hospital of Soochow University, No 303, Jingde Road, 215003 Suzhou, China

**Keywords:** Asthma, OX40, OX40L, PI3K/AKT, p38 MAPK, Th1, Th2, Th17, Treg

## Abstract

**Background:**

The aim of this study was to investigate the mechanisms of OX40L in regulating helper T (Th) cells differentiation through phosphoinositide 3-kinase (PI3K)/AKT and p38 mitogen-activated protein kinase signaling pathway in vitro and in vivo experiments.

**Methods:**

Serum samples of patients with asthma and healthy controls were used to explore the association between OX40L and Th cells. Enzyme-linked immunosorbent assay (ELISA) was used to measure the serum concentrations of OX40L, IL-4, IFN-γ, IL-17 and TGF-β. Flow cytometry method was used to analyze Th1, Th2, Th17 and Treg cells. 3H-thymidine was used to determine the proliferation of T cells. Western Blot was used to detect protein expression and phosphorylation. Immunohistochemistry was used to detect the expression of OX40L in lung tissues.

**Results:**

OX40L, IL-4, IL-17 increased in patient serum compared to healthy control and in the ovalbumin (OVA)-primed mononuclear cells compared to normal cells, while IFN-γ and TGF-β were decreased. Besides, the OVA-primed CD4^+^ T cells treated with OX40L-Ig fusion protein promoted the proliferation of T cells and Th2 and Th17 cells differentiation as well as PI3K/AKT and p38 MAPK signaling pathway, but suppressed Th1 and Treg cells differentiation. Moreover, helper T cells differentiation in OVA-primed CD4^+^ T cells could be markedly reversed by the addition of PI3K/AKT inhibition, p38 MAPK inhibition and anti-OX40L monoclonal antibody.

**Conclusions:**

In this study, we revealed that OX40L could regulate differentiation of helper T cells via PI3K/AKT and p38 MAPK signaling pathway in asthma. Besides, blockade of OX40/OX40L could inhibit the proliferation of CD4^+^ T cells and regulate polarization of helper T cells.

**Electronic supplementary material:**

The online version of this article (10.1186/s12967-018-1436-4) contains supplementary material, which is available to authorized users.

## Background

Asthma is allergic disease characterized by symptoms of variable chronic airway inflammation, airflow limitation, and airway hyperresponsiveness [1]. The exact pathogenesis of asthma is poorly understood. For many years, some studies have suggested that dysfunction of helper T cells plays a key role in allergic asthma, OX40L–OX40 interactions contribute to the differentiation of helper T cell and the maintenance of balance in asthma [2]. The OX40L (CD252, TNFSF4) which was originally termed glycoprotein 34 kDa (GP34) belongs to TNF superfamily, and it mainly expressed on the surface of antigen-presenting cells (APC), including activated dendritic cells (DCs), B cells, macrophages, T cells as well as endothelial cells [3, 4]. OX40 (ACT35, CD134, TNFRSF4) is constitutively expressed on cell surface of activated CD4^+^ T cells [5, 6]. It can specially bind to OX40L and initiate a series of reactions which contribute to facilitate the proliferation and survival of CD4^+^ T cells and cytokine secretion [7].

The imbalance and function of T-helper type Th1/Th2 were perceived as the crucial pathogenesis of asthma [8]. Under normal circumstances, mutual inhibition between Th1 and Th2 cells is conducive to the balance of immune response in the body [9, 10]. However, Th2-type immune response is mediated by cytokines including IL-4 will facilitate the occurrence of the aeroallergen and hyperresponsiveness [10]. Th2 cells can increase the secretion of Interleukin-4 (IL-4) and antagonize the emergence of Th1 cells including interferon-γ (IFN-γ) and hence further promote the development of asthma [11]. There were also some studies recognized that imbalance of Th1/Th2 does not fully account for the aetiology of asthma, and other CD4^+^ T cell subsets may be involved in asthma, including Th17 cells and regulatory T cells (Treg) [12]. The imbalance between Th17 and Treg could play a crucial role in allergic airway inflammation [13]. It has been known that Th17 cells also play significant roles in airway hyperresponsiveness and typically promote neutrophilic inflammation in accordance with Th2 cells [14]. On the other hand, Treg cells regulate immune responses to allergens and maintain immune homeostasis by preventing excessive inflammatory responses [15]. Therefore, OX40/OX40L plays a crucial role in regulating the balance of Th1/Th2 and Th17/Treg in asthma [16].

Moreover, recent studies have shown that the pathogenesis of asthma was connected with certain molecules and transcription factors [17]. Research has shown that the PI3K/AKT signaling pathway might play an vital role in regulating the proliferation of airway smooth muscle cell in asthma [18], and antagonism of the PI3K/AKT signaling pathway might be a potentially available strategy for the therapeutic intervention of asthma [19]. LY294002, a specific PI3K inhibitor, is able to significantly downregulate Akt phosphorylation and suppress inflammatory cell infiltration, mucus production and airway hyperresponsiveness in a murine asthmatic model [20]. Intratracheal administration of LY294002 to the asthmatic mice was capable of reducing airway inflammation. Besides, p38 MAPK signaling pathway also play a significant role in allergic airway inflammation [21]. The prototypical p38 MAPK inhibitor SB203580 binds to both active and inactive p38 MAPK with high affinity and inhibited p38 MAPK-mediated airway smooth muscle synthetic function [22]. Thus, the receptor signaling system in asthma regulates the effects of cytokines, adhesion factors and inflammatory mediators.

Furthermore, recent research showed that the interactions of OX40/OX40L could contribute to an ongoing immune response through the regulation of Th1 and Th2 cells involved signaling pathways, such as PI3K/AKT, nuclear transcription factor κB (NF-κB) and nuclear factor of activated T cells [23, 24]. However, little is known regarding OX40L-mediated intracellular signaling or itsmechanism in asthma by far. Hence, in this study, we aim to verify whether OX40L could induce differentiation of helper T cells via PI3K/AKT and p38 MAPK signaling pathway by using a highly specific PI3K/AKT inhibitor (LY294002) and p38 MAPK inhibitor (SB203580), respectively.

## Methods

### Detection of serum samples

This study was approved ethically by Children’s Hospital of Soochow University and written informed consent was obtained from every participant. Serum samples were obtained from 15 patients with acute episode of asthma and 15 patients with stable asthma. 15 healthy persons were served as the control group. The OX40L, Th1, Th2 and Th17 cytokines (IL-4, IFN-γ and IL-17) as well as Treg cytokines (TGF-β) in serum were measured using enzyme-linked immunosorbent assay (ELISA) kits (Sigma-Aldrich, USA) according to the manufacturer’s protocol.

### Detection of the expression of OX40L from sensitized cells

Peripheral blood samples from mice were used to isolate peripheral mononuclear cells by a Percoll (Solarbio, Beijing, China) density gradient centrifugation. The mononuclear cells stimulated with ovalbumin (OVA, chicken egg albumin, Sigma-Aldrich, USA) or phosphate buffered saline (PBS) (PBS was served as control) for 48 h. IL-4, IFN-γ and IL-17 as well as TGF-β in supernatants were measured using ELISA kits (Sigma-Aldrich, USA). The expression of OX40L on the cell surface of harvested cells was also detected by ELISA kits (Sigma-Aldrich, USA).

### Protein phosphorylation assay

Protein extracts of OVA-treated cells were prepared in a lysis buffer. Then subjected to sodium dodecyl sulfate–polyacrylamide gel electrophoresis (SDS-PAGE), transferred onto nitrocellulose membranes (Millipore, Bedford, MA, USA). Membrane were blocked for 2 h and incubated with primary antibody against p-AKT, AKT, p-p38, p38, or GAPDH (Cell Signaling, Danvers, MA, USA), followed by incubation with the HRP-conjugated secondary antibody. The bands were detected using an ECL reagent (Bio-Rad) and the quantities of the target bands were normalized by GAPDH.

### OX40L impact on helper T cells differentiation

Peripheral blood mononuclear cells (PBMC) were isolated as described previously [25]. For the PBMC preparations, CD4^+^ T cells was isolated and purified by automated magnetic sorting and anti-CD4 microbeads isolation Kits (Miltenyi Biotec) according to the manufacturer’s protocol. All sorted cell populations exhibited 95% purity, as revealed by flow cytometry (Additional file 1).

The OVA-primed CD4^+^ T cells were, respectively incubated with OX40L-Ig fusion protein or control IgG for 48 h. To estimate proliferation of T cells, 3H-TdR was added during the last 12 h of a 48 h culture [26]. Cells were harvested and counted with liquid scintillation counting (Aloka Lsc-lb7, shanghai, china). IL-4, IFN-γ, IL-17 and TGF-βlevels in supernatants were measured by ELISA according to the manufacturer’s protocol.

Flow cytometric analysis of Th1, Th2 and Th17 was performed using FITC anti-CD4 (Biolegend, San Diego, CA, USA), PE anti-IFN-γ (Biolegend), PE anti- IL-4 (Biolegend), PE anti-IL-17 (Biolegend). For analysis of Treg cells, the cells were stained with FITC anti-CD4 and PE anti-CD25 (Biolegend) and then incubated with PE anti-Foxp3 (eBioscience, San Diego, CA, USA).

### Pathway of OX40L regulate helper T cells differentiation

The OVA-primed CD4^+^ T cells were, respectively incubated with OX40L-Ig fusion protein or control IgG for 48 h. The expression and phosphorylation of AKT and p38 were detected by Western blot. Moreover, after OVA-primed CD4^+^ T cells incubated with OX40L-Ig fusion protein or control IgG for 48 h, PI3K/Akt inhibitor, LY294002 or p38MAPK inhibitor, SB203580 were added. Then, 3H-TdR was added during the last 12 h of a 48 h culture. Cells were harvested and counted with liquid scintillation counting. IL-4, IFN-γ, IL-17 and TGF-β levels in supernatants were measured by ELISA according to the manufacturer’s protocol. Cells count of Th1, Th2, Th17 and Treg were measured by flow cytometry.

### OX40L impact on helper T cells differentiation in murine model

All experiments were in accordance with the ethical standards and were approved by the Soochow University. To induce experimental models of asthma, C57BL/6 mice were sensitized with OVA by intraperitoneal injection on days 0, 7 and 14, and then challenged with aerosolized 5% OVA for 30 min on days 22, 24, 26 and 28. Saline-sensitized and challenged mice were used as control [27]. To evaluate the role of OX40L, the mouse models were administered intraperitoneally with OX40L-Ig fusion protein, isotype control IgG or anti-mouse-OX40L mAb (Biolegend, USA) on days 0, 3, 7, 10 and 14 at the time of OVA sensitization. The mice were euthanized 24 h after the last challenge and lung tissues and the bronchoalveolar lavage fluid (BALF) were harvested. IFN-γ, IL-4, IL-17 and TGF-β in BALF were detected by ELISA. The number of Th cells was analyzed by FCM. In another set of experiments, the right lungs were immersed in 10% paraformaldehyde and permeabilized for histological examination containing inflammatory cell infiltration, pathological changes, and expression of OX40 and OX40L after staining with hematoxylin and eosin (H&E), periodic acid-schiff (PAS) [28] and OX40 and OX40L Immunohistochemistry, respectively.

### Statistical analysis

All data were analyzed with SPSS17.0 (SPSS Inc, USA) and expressed as the mean ± standard deviation (SD). Student’s *t* test was used to analyze differences between two groups. One-way ANOVA analysis was used to determine the multi-sample analysis. All statistical tests were two-sided, and *p* value less than 0.05 was considered significant.

## Result

### High expression of OX40L in patient serum

**Fig. 1:**
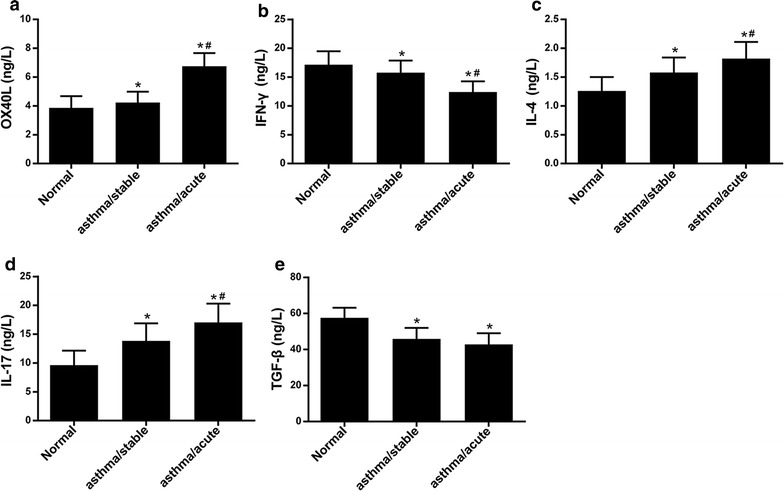
The expressions of OX40L, IFN-γ, IL-4 and IL-17 as well as TGF-β in patient serum with bronchial asthma. Normal: healthy person; asthma/stable: the patient with stable bronchial asthma; asthma/acute: the patient with acute attack of bronchial asthma; **a**–**e** The expression of OX40L, IFN-γ, IL-4, IL-17 and TGF-β. *patient with stable asthma differ significantly (p < 0.05) with normal; ^#^patient with acute attack of bronchial asthma differ significantly (p < 0.05) with patient with stable bronchial asthma

As shown in Fig. 1, the OX40L, IL-4 and IL-17 in patients with acute episode of asthma were increased in comparison with patients with stable asthma or healthy control, OX40L, IL-4 and IL-17 in patients with stable asthma were increased in comparison with healthy control. However, IFN-γ and TGF-β were decreased in patients with asthma compared with healthy control. This data suggests that OX40L plays a vital role in asthma.

### High expression of OX40L in OVA-challenged mononuclear cells

**Fig. 2:**
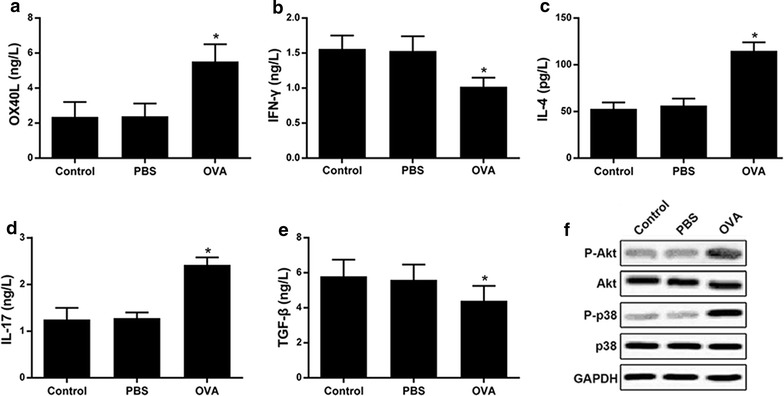
The expressions of OX40L, IFN-γ, IL-4 and IL-17 as well as TGF-β in mononuclear cells. Control: the normal mononuclear cells; PBS: the mononuclear cells stimulated with PBS; OVA: the mononuclear cells stimulated with OVA. **a**–**e** The expression of OX40L, IFN-γ, IL-4 and IL-17 and TGF-β. **f** The phosphorylation and protein expressions levels of Akt, p38 MAPK and GAPDH using Western blot. *p < 0.05 versus control group

As shown above, OX40L was highly expressed in patient with asthma. Thus, we detected the expression of OX40L in OVA-challenged mononuclear cells and the results showed that the expression of OX40L was higher in OVA group compared with PBS control (Fig. 2a). Similarly, the expression of IL-4 and IL-17 were also higher in OVA group compared with PBS control (Fig. 2c, d). By contrast, IFN-γ and TGF-β were decreased in OVA group in comparison with PBS control (Fig. 2b, e). In addition, the level of phosphorylation of AKT and p38 MAPK were elevated in OVA group (Fig. 2f).

### OX40L induce helper T cells differentiation through PI3 K/AKT and p38 MAPK signaling

**Fig. 3:**
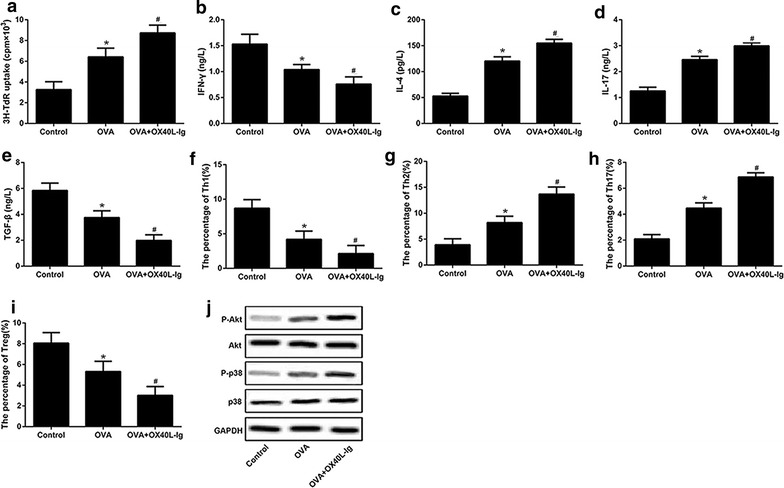
The effect of OX40L-Ig fusion protein on proliferation and differentiation of CD4^+^ T cells. Control: the OVA-primed CD4^+^ T cells; OVA: the OVA-primed CD4^+^ T cells treated with Ig; OVA + OX40L-Ig: the OVA-primed CD4^+^ T cells treated with OX40L-Ig fusion protein. **a** The proliferation of CD4^+^ T cells. **b**–**e** The expression of OX40L, IFN-γ, IL-4 and IL-17 and TGF-β. **f**–**i** Cells count of Th1, Th2, Th17 and Treg. **j** The phosphorylation and protein expressions levels of Akt, p38 MAPK and GAPDH using Western blot.*OVA + OX40L-Ig group differ significantly (p < 0.05) with control. ^#^OVA + OX40L-Ig group differ significantly (p < 0.05) with OVA

As shown in Fig. 3a, the proliferation of CD4^+^ T cells was increased in OVA group as compared to the control, which was further increased when treated with OX40L-Ig fusion protein. The level of IL-4 (Fig. 3c) and IL-17 (Fig. 3d) were increased and the level of IFN-γ (Fig. 3b) and TGF-β (Fig. 3e) were decreased in OVA group and OVA + OX40L-Ig group. Similarly, the number of Th2 (Fig. 3g) and Th17 (Fig. 3h) were increased and the number of Th1 (Fig. 3f) and Treg (Fig. 3i) were decreased in OVA group and OVA + OX40L-Ig group compared to control group. OX40L-Ig fusion protein enhanced the effect of OVA challenge on Th cell subtypes (Additional file 1). In addition, as shown in Fig. 3j, we found that the level of phosphorylation of AKT and p38 MAPK were elevated in OVA-challenged CD4^+^ T cells, and more elevated in OVA-challenged CD4^+^ T treated with OX40L-Ig fusion protein. The above results suggest that PI3K/AKT and p38 MAPK signaling pathways may be involved in the OX40L induced differentiation of helper T cells.

**Fig. 4:**
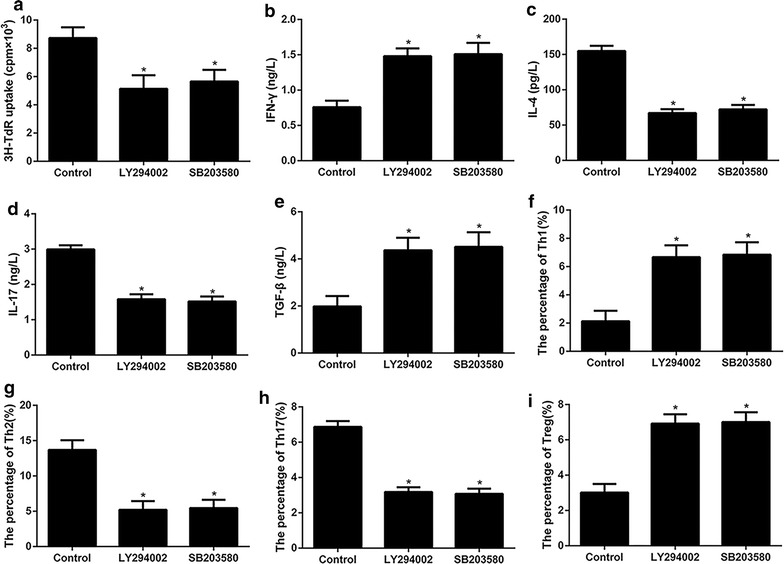
The role of PI3K/Akt and p38 MAPK signaling pathway in OX40L induced proliferation and differentiation of CD4^+^ T cells. Control: the OVA-primed CD4^+^ T cells treated with OX40L-Ig fusion protein; LY294002 or SB203580: the OVA-primed CD4^+^ T cells treated with OX40L-Ig fusion protein and then incubated with PI3 K/Akt inhibitor, LY294002or p38MAPK inhibitor SB203580, respectively. **a** The proliferation of CD4^+^ T cells. **b**–**e** The expression of IFN-γ, IL-4 and IL-17 and TGF-β. **f**–**i** Cells count of Th1, Th2, Th17 and Treg. *p < 0.05 versus control

Accordingly, we next investigated whether PI3K/Akt inhibitor, LY294002 or p38MAPK inhibitor, SB203580 would have a suppressive impact on helper T cells differentiation. The results shown in Fig. 4a, LY294002 and SB203580 dramatically decreased the proliferation of CD4^+^ T cells challenged by OVA. IL-4, IL-17 and the number of Th2 and Th17 cells were decreased in OVA-challenged CD4^+^ T cells after treatment of LY294002 and SB203580 (Fig. 4c, d, g, h). In contrast, the expression of IFN-γ and TGF-β as well as cells the number of Th1 and Treg were remarkably decreased by LY294002 and SB203580 (Fig. 4b, e, f, i, Additional file 2).

### OX40L induce helper T cells differentiation experiment in vivo

**Fig. 5:**
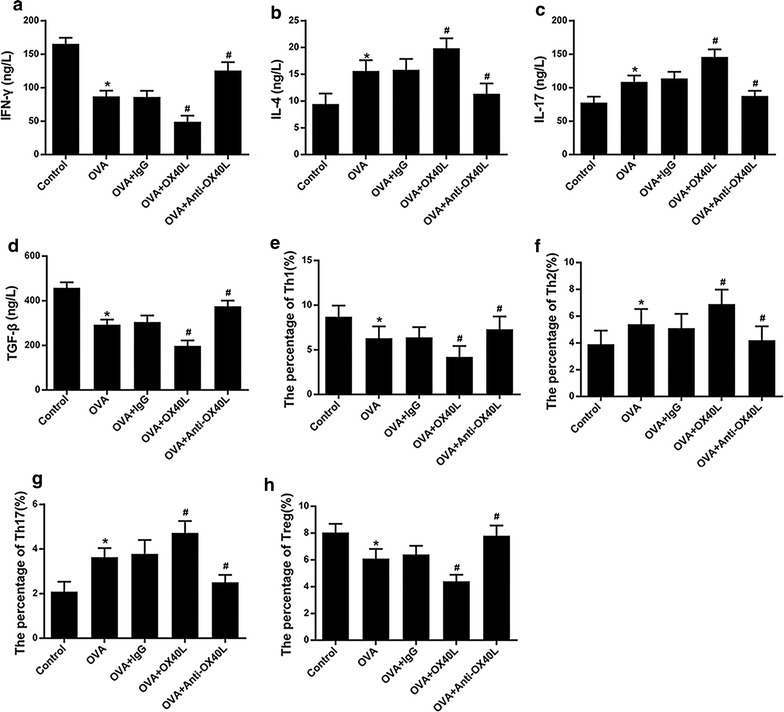
The effect of OX40L-Ig fusion protein and anti-mouse-OX40L mAb on Th cells differentiation in asthma models. Control: challenged mice with Saline; OVA: sensitized and challenged mice with OVA; OVA + IgG: OVA-primed mice treated with IgG; OVA + OX40L: OVA-reduced mice treated with OX40L-Ig fusion protein; OVA + anti-OX40L: OVA-reduced mice treated with anti-mouse-OX40L mAb. **a**–**d** The expression of IFN-γ, IL-4 and IL-17 and TGF-β. **e**–**h** Cells count of Th1, Th2, Th17 and Treg. *p < 0.05 versus control, ^#^p <  0.05 versus OVA

**Fig. 6:**
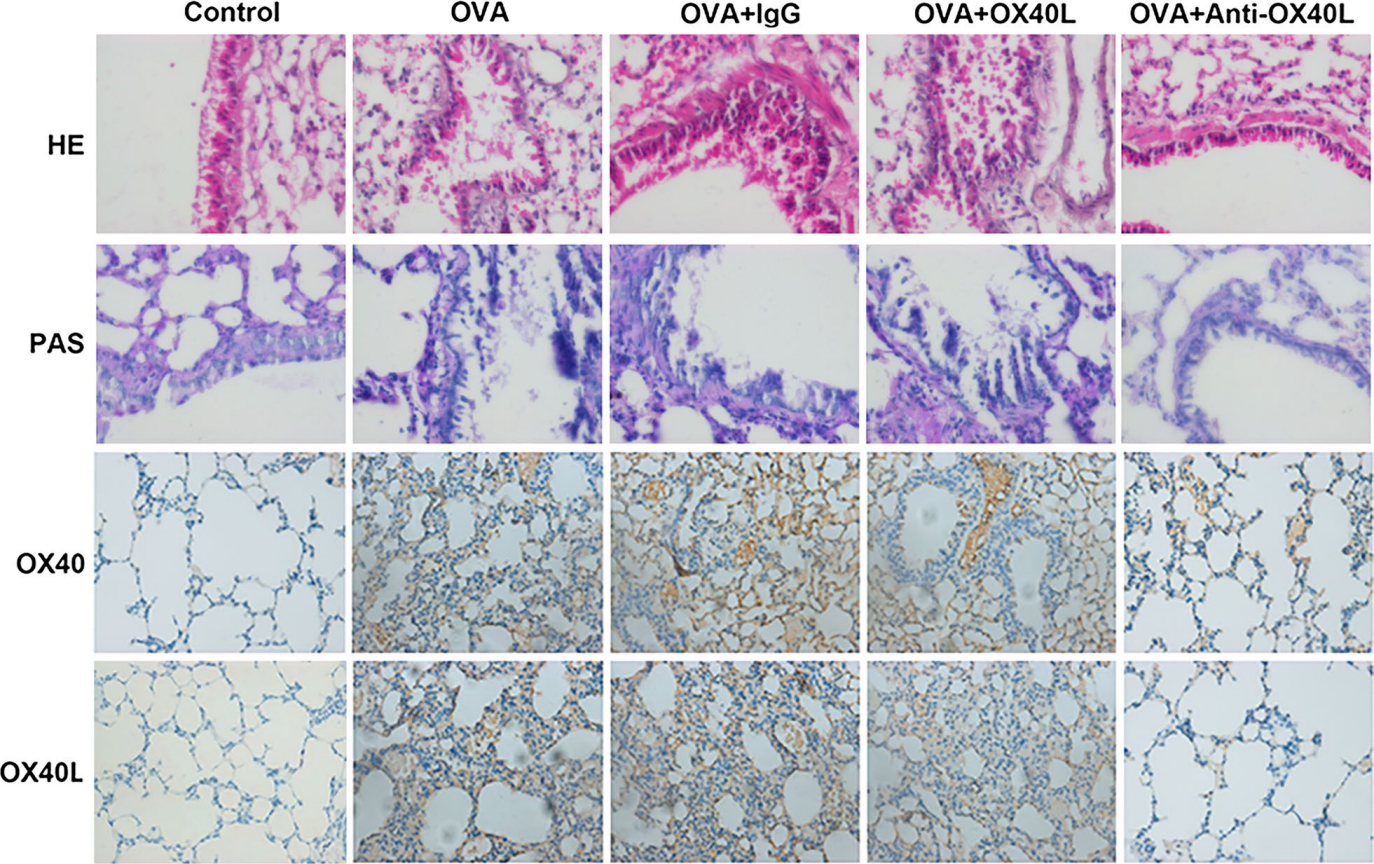
Immunohistochemistry analysis of asthma models treated with OVA, OX40L-Ig fusion protein and anti-mouse-OX40L mAb. More significantly increased inflammatory cells infiltration and expression of OX40L in OVA + OX40L, and infiltration of eosinophils in the lung tissue was markedly decreased in OVA + anti-OX40L

To identify the role of OX40L in differentiation of helper T cells, murine model of asthma was established. The results shown in Fig. 5, Additional file 3, IL-4, IL-17 and the number of Th2 and Th17 cells were significantly increased while IFN-γ and TGF-β as well as the number of Th1 and Treg were significantly decreased in OVA-challenged mice treated of OX40L-Ig fusion protein when compared with the control group and OVA group. On the contrary, IL-4, IL-17 and the number of Th2 and Th17 cells were significantly decreased when treatment of the anti-OX40 mAb, while the IFN-γ and TGF-β as well as the number of Th1 and Treg were significantly increased. These results indicate that absence of OX40L could regulate the differentiation of helper T cells. To determine the effects of OX40L-Ig fusion protein and anti-OX40 mAb on histological lung inflammation, HE staining and PAS staining were used to measure airway inflammation and mucus production. As expected, the results in Fig. 6 showed that there were prominently increased inflammatory cells infiltration, particularly of lymphocyte and eosinophils in the perivascular and peribronchial spaces, thickening of the basement layer below the epithelium in OVA-challenged mice and OVA-challenged mice treated with IgG. Besides, more significantly increased inflammatory cells infiltration and expression of OX40L in OVA-challenged mice treated with OX40L-Ig fusion protein. On the contrary, in OVA-challenged mice treated with anti-OX40 mAb, infiltration of eosinophils in the lung tissue was markedly decreased as well as the basement layer epithelium thickening. Therefore, the histological examination demonstrated that OX40L played a crucial role in inducing helper T cells differentiation in asthma (Additional file 3).

## Discussion

Bronchial asthma is a type of airway allergic inflammation disease caused by variety of inflammatory cells and mediators. Inflammatory cells and released mediators played an important part in the complicated pathogenesis of asthma [29, 30]. Thereby, in this article, we principally focus on the mechanism of OX40L in asthma. In our study, we found that OX40L was significantly higher in patients with asthma, suggesting the possibility that OX40L was associated with asthma. There were a few articles have confirmed this point, such as, Ezzat et al. found the mean and median of OX40L levels were dramatically higher in asthmatic children during acute attacks children than in children with moderate mild asthma exacerbations [31] and Siddiqui et al. showed the number of OX40, OX40L and IL-4 were significantly increased in subjects with mild asthma compared with healthy controls [1]. Moreover, we also found that the OX40L was increased in mononuclear cells stimulated with OVA, suggesting that OX40L played a significant role in the asthma response induced by helper T cells. Specifically, OX40/OX40L interactions played a potential role in differentiation of T cells and has an influence on the balance of Th1/Th2 [32]. Note that OX40L can activate the expression of IL-4 by T cell, inhibit the expression of IFN-γ and participate in the immunocyte including mastocyte mediated Th2-type immune response [33]. In addition, the expression of OX40 induced by Th17 cells and signaling through OX40 also contributes to Th17-type immune response [34] and costimulatory receptor OX40L can prevent differentiation of Tregs and block their function [35]. Moreover, the signaling pathways involved in asthma, for instance, tyrosine kinase signaling cascades played a vital role in allergic airway inflammation [36], and GM-CSF activation of STAT5 pathway delayed apoptosis of lung granulocytes in this asthma [37]. In addition, TGF-beta signaling was active in asthmatic airways and the activity was associated with the development of airway remodeling in asthma [38]. Similarly, PI3K/AKT and MAPK were tightly associated with asthma. Various studies have shown that PI3K/AKT pathway was linked to the development of asthma and inhibition of PI3K/AKT signaling might attenuate allergic asthma [39]. It has also been demonstrated that p38 MAPK pathway regulated the expression of IL-4 and IL-5 at the levels of mRNA and protein, which played a crucial role in the pathogenesis of asthma [40]. Subsequently, we examined whether preventive effect of PI3K/AKT and p38 MAPK inhibition on OX40L in asthma could be via PI3K-AKT and p38 MAPK pathway. Our results demonstrate that OX40L significantly activated PI3K, AKT and P38 MAPK protein kinases in asthma which were significantly prevented by inhibitors of PI3 K and p38 MAPK. These observations suggest that PI3K/AKT and p38 MAPK would have a suppressive impact on differentiation of helper T cells and OX40L induced differentiation of helper T cells through PI3K/AKT and p38 MAPK signaling pathway.

Furthermore, we investigated the role of OX40L in asthma using a mouse asthma model in vivo, and the interaction of OX40/OX40L indeed tightly associated with asthma in differentiation of helper T cells. This study provides a more innovative and comprehensive understanding of OX40/OX40L in regulating differentiation of helper T cells in asthma, which also provide novel insight for asthma prevention and possible target for drug development.

## Conclusions

In summary, our results from the present studies demonstrated that OX40L induced differentiation of helper T cells via PI3K/AKT and p38 MAPK pathway and blockade of OX40L with anti-OX40L mAb could inhibit the differentiation of helper T cells in chronic asthma.

## Additional files

**Additional file 1.** Flow cytometric analysis of Th1, Th2, Th17 and Treg in OVA and OX40L-Ig fusion protein treated CD4^+^ T cells.

**Additional file 2.** Flow cytometric analysis of Th1, Th2, Th17 and Treg in OVA-challenged CD4^+^ T cells treated with OX40L-Ig fusion protein and PI3K/Akt inhibitor, LY294002 or p38MAPK inhibitor SB203580.

**Additional file 3.** Flow cytometric analysis of Th1, Th2, Th17 and Treg in OVA, OX40L-Ig fusion protein or anti-mouse-OX40L mAb treated CD4^+^ T cells.

## Abbreviations

3H-TdR: 3H-thymidine
DCs: dendritic cells
ELISA: enzyme-linked immunosorbent assay
FCM: flow cytometry method
H&E: hematoxylin and eosin
IL-4: interleukin-4
IFN-γ: γ-interferon
mAb: monoclonal antibody
NF-κB: nuclear transcription factor κB
OX40L: OX40 ligand
OVA: ovalbumin
PI3K: phosphoinositide 3-kinase
P38 MAPK: p38 mitogen-activated protein kinase
PAS: periodic acid-schiff
Treg: regulatory T cells
