# Supplementary figures and images for: OX40L induces helper T cell differentiation during cell immunity of asthma through PI3K/AKT and P38 MAPK signaling pathway
Source: J Transl Med. 2018 Mar 20;16:74. doi: 10.1186/s12967-018-1436-4 (PMC5859438; doi:10.1186/s12967-018-1436-4)

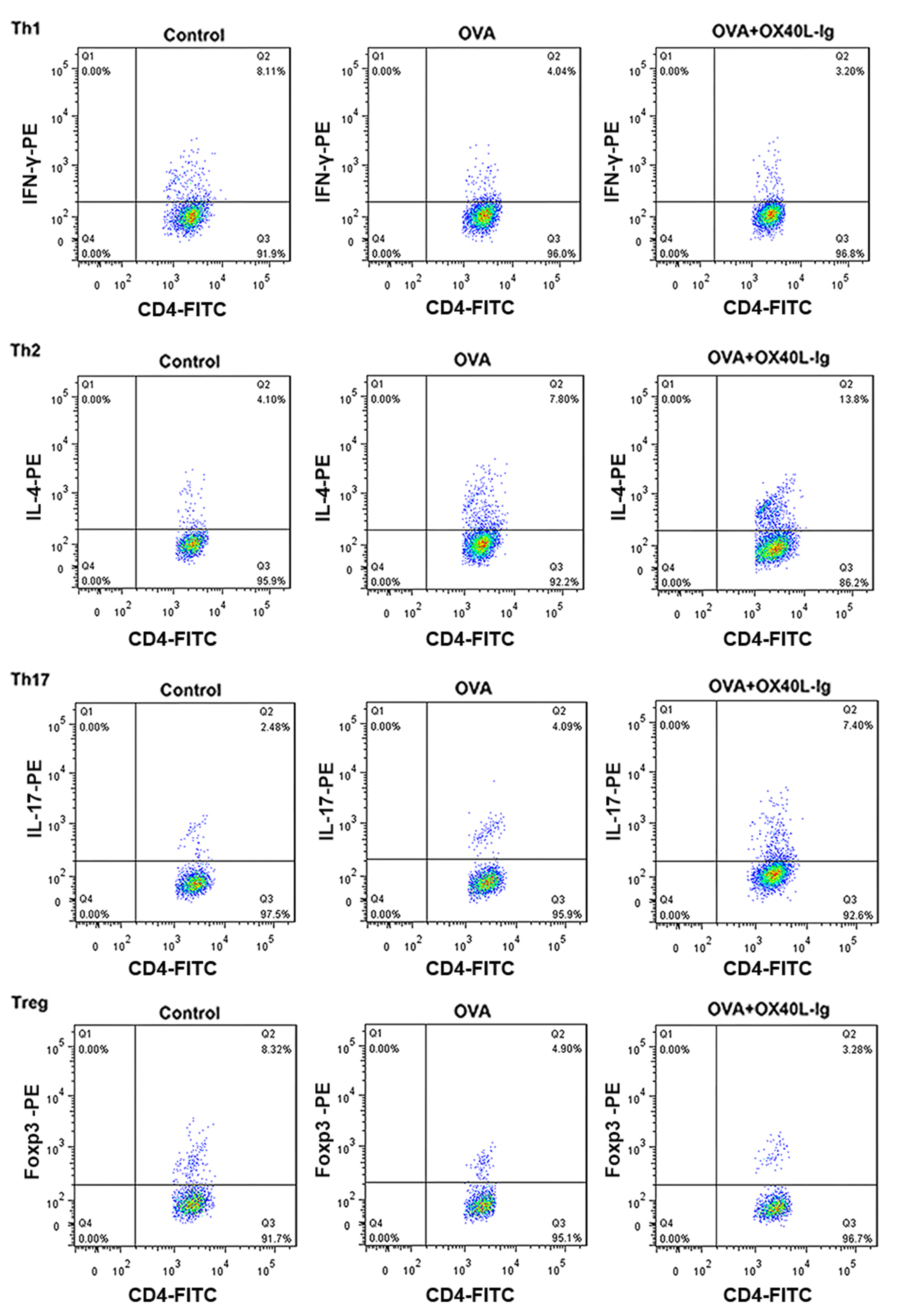

Supplement: Supplementary file 1 — Additional file 1. Flow cytometric analysis of Th1, Th2, Th17 and Treg in OVA and OX40L-Ig fusion protein treated CD4+ T cells. [file 12967_2018_1436_MOESM1_ESM.tif]

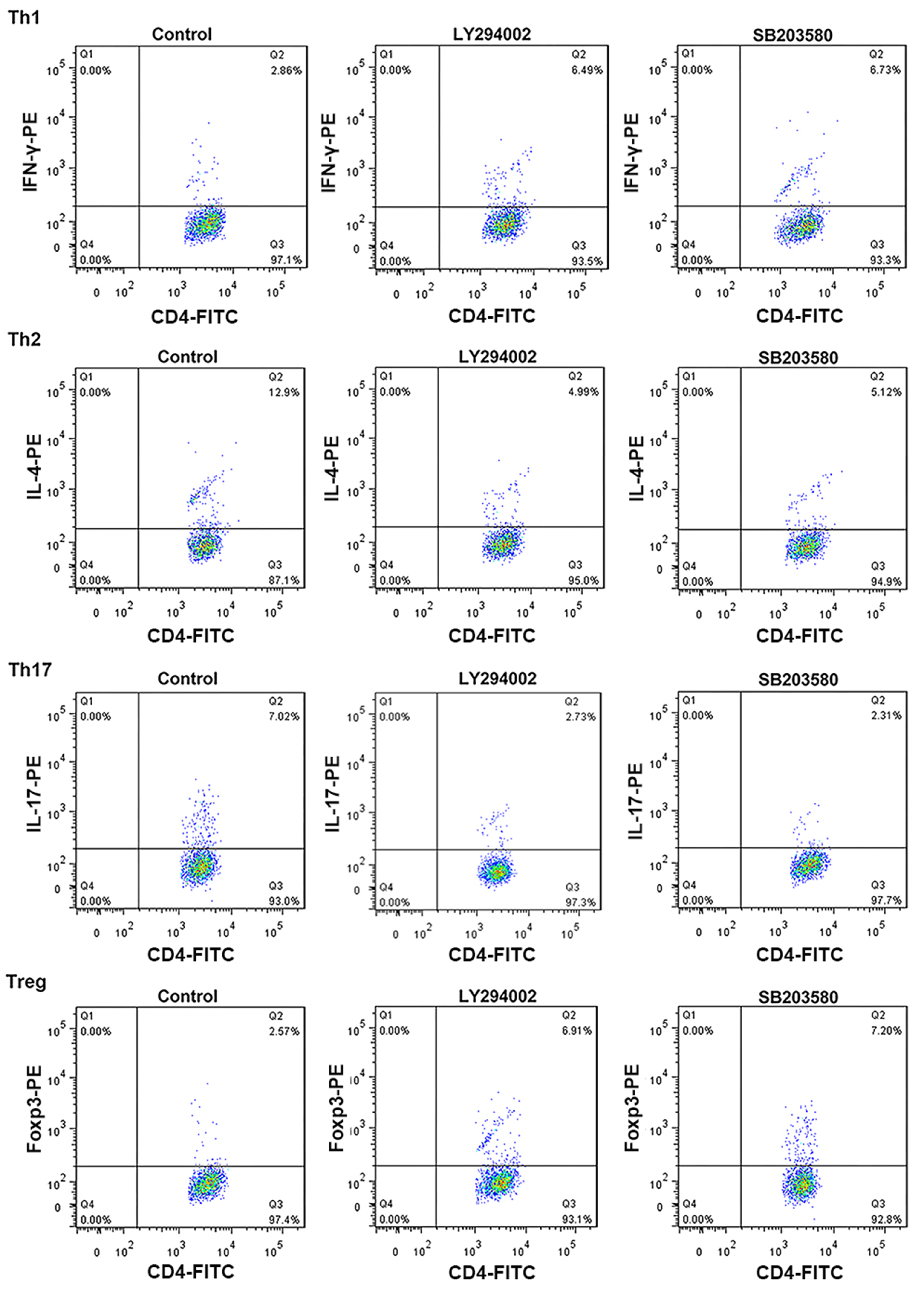

Supplement: Supplementary file 2 — Additional file 2. Flow cytometric analysis of Th1, Th2, Th17 and Treg in OVA-challenged CD4+ T cells treated with OX40L-Ig fusion protein and PI3K/Akt inhibitor, LY294002 or p38MAPK inhibitor SB203580. [file 12967_2018_1436_MOESM2_ESM.tif]

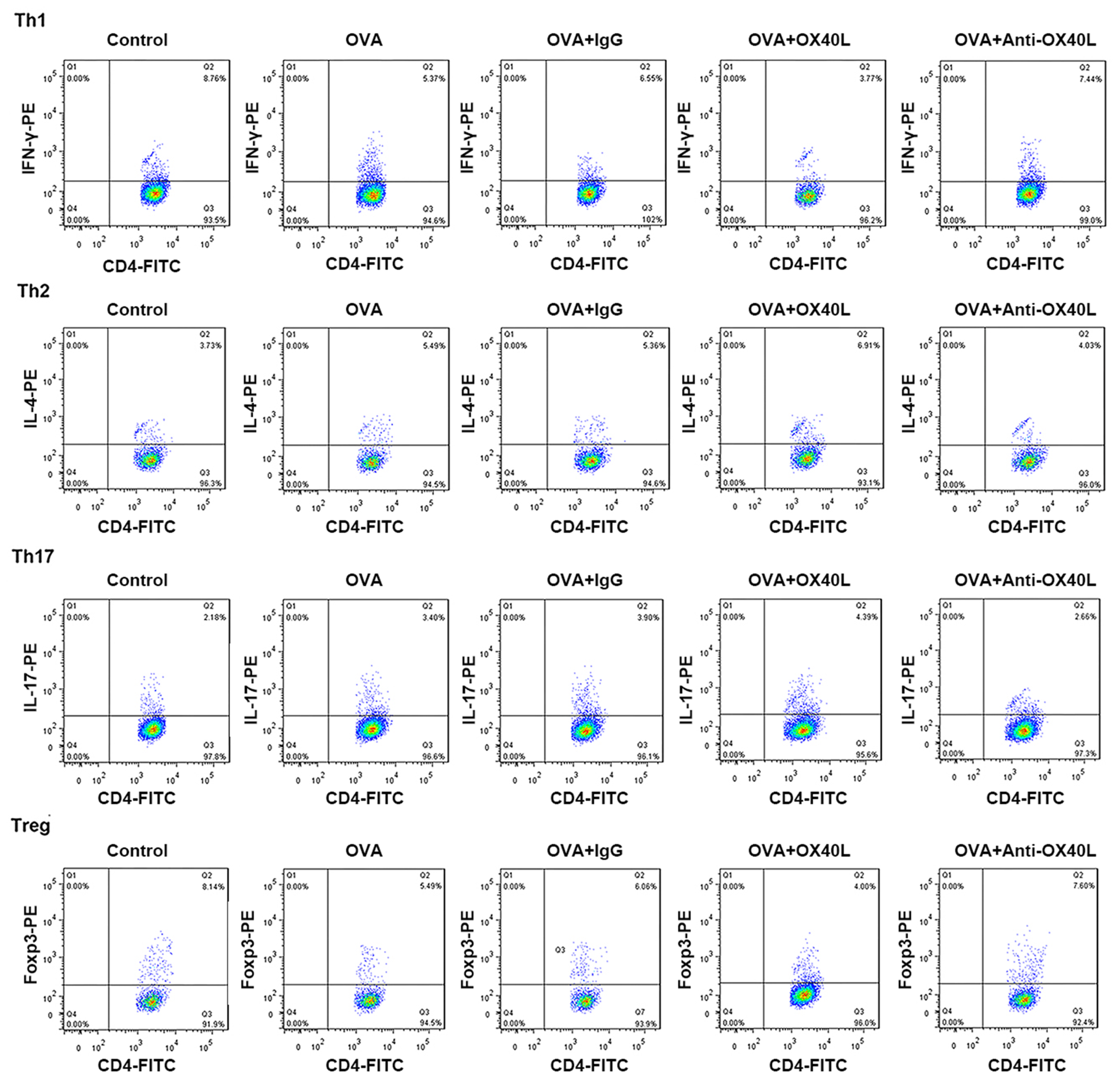

Supplement: Supplementary file 3 — Additional file 3. Flow cytometric analysis of Th1, Th2, Th17 and Treg in OVA, OX40L-Ig fusion protein or anti-mouse-OX40L mAb treated CD4+ T cells. [file 12967_2018_1436_MOESM3_ESM.tif]
